# B cell responses to apoptotic cells in MFG-E8-/- mice

**DOI:** 10.1371/journal.pone.0205172

**Published:** 2018-10-04

**Authors:** YuFeng Peng

**Affiliations:** Division of Rheumatology, Department of Medicine, University of Washington, Seattle, Washington, United States of America; Instituto Nacional de Ciencias Medicas y Nutricion Salvador Zubiran, MEXICO

## Abstract

Defective clearance of apoptotic cells in MFG-E8 deficient mice results in lupus-like disease in the mixed B6x129, but not pure B6 background. The lack of overt autoimmunity in MFG-E8-/- B6 mice suggests that accumulation of apoptotic cells is not sufficient to break central tolerance. However, the delayed clearance of apoptotic cells in the follicles of MFG-E8-/- B6 mice provides an excellent opportunity to investigate how B cells respond to excessive apoptotic cells in the periphery under relatively non-inflammatory conditions. In MFG-E8-/- B6 mice, we found increased IgG2c production against apoptotic cells and oxidized LDL. Apoptotic cell induced antibody responses depended on MyD88 signal and T cell help. In addition, MFG-E8-/- B6 mice had enlarged MZ B cell compartments as well as an enhanced antibody response to NP-Ficoll. Moreover, a significant percentage of MZ B cells in aged MFG-E8-/- B6 mice migrated into follicles. Injecting apoptotic cells or oxidized LDL into wild type mice as well as physiological accumulation of LDL in ApoE-/- mice recapitulated the translocation of MZ B cells. To determine how MFG-E8 deficiency affects the functions of autoreactive B cells specific for nucleic acids in the periphery under non-inflammatory conditions, we utilized BCR transgenic mice to bypass central selection and compared the differentiation of TLR9 dependent anti-dsDNA 56R B cells and TLR7 dependent anti-ssRNA H564 B cells in MFG-E8-/- mice. In MFG-E8-/- 56R mice, anti-dsDNA specific 56R/Vκ38c B cells differentiated into MZ B cells but not AFCs. On the contrary, in MFG-E8-/-H564 mice, anti-ssRNA specific H564 B cells further differentiated into GC B cells and AFCs. Adoptive transfer of activated autoreactive B cells confirmed that H564 B cells were more sensitive to apoptotic cell antigens than 56R B cells. Our observations provide new insights about the MZ B cell translocation in lupus patients as well as the dichotomy of TLR9 and TLR7 signals in the pathogenesis of lupus.

## Introduction

Both central and peripheral tolerance play critical roles in controlling autoreactive B cells [1]. Most antibodies encoded by the germline are autoreactive. In bone marrow, autoreactive immature B cells are either deleted, forced to undergo receptor editing, or become anergic. Once they arrive in the periphery, mature B cells can re-acquire auto-reactivity through somatic mutation during GC (germinal center) reaction. Antigens deposited on FDCs (follicular dendritic cells) in the GC play an important role in selecting mutated B cells: B cells with the highest affinities differentiate into memory cells, whereas those with low affinities, including potentially autoreactive clones are deleted. The selection of autoreactive B cells depends on the threshold of B cell activation. Numerous animal models have demonstrated that defects in both central and peripheral B cell tolerance are required to develop overt lupus-like disease[2].

Spleen B cells consist of two major populations: MZ (marginal zone) B cells and FO (follicular) B cells. Under normal conditions, MZ B cells and FO B cells are separated by the marginal zone, which also includes various types of macrophages. Because of their location, marginal zone macrophages and B cells are the first line to capture and to respond to circulating antigens. An intact marginal zone is required to maintain an effective defense against both foreign and self antigens. Consistent with their innate-like immunity, the antibody repertoire of marginal zone B cells is enriched in poly-reactivity[3–5]. Moreover, marginal B cells also shuttle between the marginal zone and follicles to deposit antigens on FDCs[6]. Disrupting this shuttling through a S1P1 antagonist prevented optimal antibody responses [6]. In lupus patients, autoreactive 9G4+ B cells migrated into follicles [7], suggesting MZ B cells in lupus patients may be more facile in transporting auto-antigens and they may also directly participate in GC reactions. The signals that drive MZ B cell translocation in lupus patients have not been identified.

In the well-established HEL model system, how antigens are presented determines the fate of HEL specific B cells [8]. Recent studies suggest similar mechanisms may also apply to bona fide self-reactive B cells. Self antigens *in vivo* are associated with apoptotic cells. The lipid components of apoptotic cell membranes are oxidized [9]. These oxidized lipids, to some degree similar to the lipid found on surface of bacteria, provide neo-antigens to stimulate innate B cell responses [10]. Moreover, apoptotic blebs on the surface of apoptotic cells contain both DNA and RNA fragments [11, 12]. The elegant study by Leadbetter et al. demonstrated, these DNA and RNA fragments could form immune complexes with autoantibodies to provide endogenous TLR9 and TLR7 ligands thereby activating AM14 B cells [13]. However, because AM14 B cells are specific to IgG2a rather than self-antigen, how bona fide autoreactive B cells respond to apoptotic cells remains to be determined.

Numerous mouse studies also demonstrated the dichotomy of TLR9 and TLR7 in lupus pathogenesis. While genetic deletion of TLR9 reduced the production anti-dsDNA, it exacerbated disease. On the contrary, deletion of TLR7 inhibited the production of anti-ssRNA antibody and prevented lupus [14]. These contrasting effects between TLR9 and TLR7 signals may reflect the different pathogenic potentials between anti-dsDNA and anti-ssRNA antibodies or different B cell responses to apoptotic cell associated nucleic acid antigens.

Not only do apoptotic cells provide antigens to stimulate self-reactive B cells [11], they also accumulate in lupus patients. Macrophages derived from lupus patient PBMC (peripheral blood mononuclear cells) are defective in phagocytosing apoptotic cells, and un-ingested fragments of apoptotic cells were found in the germinal centers of a subset of lupus patients [15, 16]. Clearance of apoptotic cells is mediated through various opsonins[17]. MFG-E8, a soluble protein that bridges the PS (phosphatidylserine) on apoptotic cells and integrins on macrophages, can facilitate the removal of apoptotic cells [18]. MFG-E8 is expressed by myeloid cells and tangible body macrophages in the follicle. In the absence of MFG-E8, apoptotic cell debris can be found on the surface of macrophages during the GC reaction [18]. Therefore, the MFG-E8-/- mouse model approximates the defective clearance of apoptotic cells in lupus patients.

Although bolus injection of exogenous apoptotic cells has been commonly used to investigate immune responses against apoptotic cells, most of the injected materials are trapped within marginal zone macrophages and excluded from the follicle and FDCs ([19] and personal observations). Even after repeated injections, exogenous apoptotic cells without adjuvant rarely induce IgG responses in w.t. mice [20]. The inability to access FDCs may explain their ineffectiveness. To mimic the physiological accumulation of apoptotic cells within the follicle and on FDCs in lupus patients, we chose MFG-E8-/- mice as a model to determine how apoptotic cell accumulation affects the differentiation of B cells in the periphery. To avoid the defective central tolerance associated with autoimmune prone B6x129 mice and the inflammatory condition associated with autoimmune diseases, we used non-autoimmune MFG-E8-/- B6 mice in this study to investigate how autoreactive B cells react to excessive apoptotic cells in the periphery under relatively non-inflammatory conditions.

## Material and methods

### Mice

MFG-E8-/- mice in a B6 background were from Dr. Nagata (Kyoto University, Japan). 56R anti-dsDNA BCR (B cell receptor) transgenic mice in a B6 background were from Dr. Prak (University of Pennsylvania). H564 anti-ssRNA B cell receptor transgenic mice in a B6 background were originally from Dr. Imanishi-Kari (Tufts University) and kindly provided by Dr. Keith Elkon (University of Washington). TCRα-/-, MyD88-/-, ApoE -/-, and LDLR-/- mice in a B6 background were purchased from Jackson Laboratory (Maine). All animal (mice) usage and procedures in this study were carried out in strict accordance with the recommendations in the Guide for the Care and Use of Laboratory Animals of the National Institutes of Health. The protocol was approved by the Institutional Animal Care and Use Committee of the University of Washington (IACUC Protocol Number: 3380–02). No surgery was performed in this study. Death of animal was not used as outcome or experimental endpoint in this study. All mice were euthanized with CO_2_. The number of animals used in each study was estimated using the following parameters: number of tails = 1, effect size = 2, α = 0.05 and power = 0.8.

### Antibodies

Antibodies against F4/8 (A3-1), CD169 (3D6.112), MADCAM (MECA-367) were from Serotec. FITC conjugated polyclonal goat anti-mouse C3 was from MP Biomedicals. HRP conjugated goat anti-mouse IgM, IgG, IgG1, IgG2b, IgG2c, and IgG3 were from Southern Biotech. Alexa 647 labeled anti-mouse IgG2c was from Jackson Immunoresearch laboratory. Antibodies against CD21 (7E9), CD23 (B3B4), CD1d (1B1), B220 (RA3-6B2), Ki67 (11F6), GL7 (GL7), CD1d (1B1), CXCR5 (L138D7) were from Biolegend. Fas (Jo2) antibody was from BDbiosciences. PNA was from Sigma-Aldrich. Idiotype specific antibodies against 56R/Vκ38c and 56R/Vκ21D were kind gifts from Dr. Tsubata (Japan) [21]. Idiotype specific antibody against H564 BCR was kindly provided by Dr. Imanishi-Kari [22]. Idiotype specific antibodies were biotin labeled according to the instruction of the manufacturer (Thermo Fisher Scientific).

### ELISA

MDA-LDL was purchased from Academy Bio-Medical. Two micrograms per ml MDA-LDL was used to coat ELISA plates (Nunc). Serum samples were diluted 1/100 in PBS/BSA and incubated overnight at 4°C. The binding of IgG2c was detected by HRP conjugated goat anti-IgG2c antibody. Anti-dsDNA ELISA was performed as previously described [23]. Briefly, ELISA plates were coated with 10μg/ml calf thymic DNA (Sigma) diluted in high binding buffer (Pierce). Serum samples (1/100 dilution) were incubated overnight at 4°C. The titers of anti-idiotype antibodies were measured by coating ELISA plates with 2μg/ml idiotype specific antibody overnight at 4°C. Serum samples (1/50 dilution) were incubated overnight at 4°C.

### ELISPOT

Multiscreen filter plates (Millipore) were coated with 1μg/ml idiotype specific antibody (B6.265) overnight at 4°C. The coated plates were blocked with RPMI/10%FBS for 1 hour. Serial dilutions (1/2) of spleen cells were dispensed onto the plate and cultured overnight at 37°C. IgG producing AFCs were visualized using HRP conjugated goat anti-mouse IgG antibody, followed by one-step NBT/BCIP substrate (Pierce). The number of positive cells in each well was counted using ImmunoSpot (CTL).

### B cell migration assay

Spleen cells were first plated on petri-dishes at 37°C for 30 minutes to deplete macrophages. Non-adherent cells were then re-suspended in RPMI + 10% FBS at a density of 5 x10^6^ cells/ml. CXCL13 (Peprotech) diluted in 600 μl RPMI/0.5% BSA (final concentration 800ng/ml) was added to the bottom of each well of a 24 well plate. One hundred micro liters of spleen cells were added to a 5nm pore size transwell (Corning), then placed on the top of the well. After a 4 hour incubation at 37°C, migrated cells were collected from the bottom and enumerated by flow cytometry. MZ B cells and follicle B cells were distinguished by their expression of CD21 and CD23.

### Immunofluorescence staining

Spleen and kidney samples were snap-frozen in OCT. Five micron thick sections were stained using various antibodies. The intensity of antibody binding was quantified by image J.

### Bone marrow chimera

Recipient mice were irradiated with 1,000 rad. One day later, T and B cell depleted bone marrow cells (5x 10^6^ per mouse) were transferred to reconstitute the irradiated mice. Spleen cells and bone marrow cells from the recipients were analyzed at 3–4 month after reconstitution.

### Injection of oxidized LDL

One hundred micrograms of oxidized Hi TBAR LDL (BT- 910X, Thermo fisher) was i.v. injected into w.t. B6 mice. Forty eight hours later, spleens were collected and snap-frozen in OCT. Five micron thick spleen sections were stained with anti-CD1d to locate MZ B cells.

### Statistical analysis

In most experiments, a non-parametric student t test was used. For comparisons between multiple groups, one way A-NOVA was used. P values less than 0.05 were considered significant. * p<0.05, ** p<0.01, *** p < 0.001, **** p<0.0001, n.s. = not significant.

## Results

### Increased antibody responses to apoptotic cells in MFG-E8-/- B6 mice

**Fig 1:**
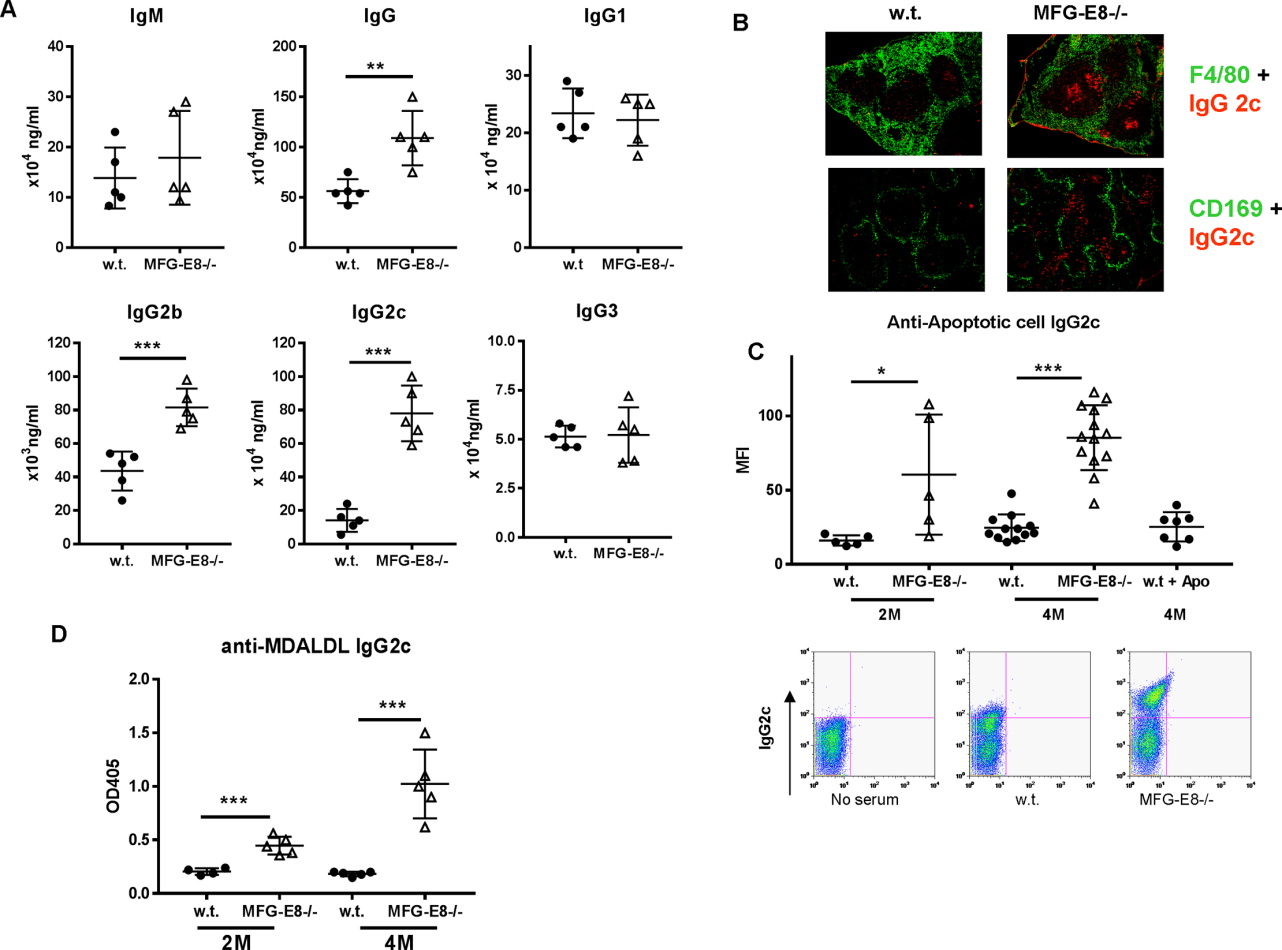
Enhanced antibody responses to apoptotic cells in MFG-E8-/- B6 mice. **A:** Total IgM, IgG, IgG1, IgG3, IgG2b, and IgG2c levels in the sera collected from 4 month old w.t. and MFG-E8-/- female mice were determined by ELISA. **B:** Localization of IgG2c+ (red) plasma cells in the spleens of 4 month old w.t. and MFG-E8-/- mice was examined by immunofluorescence staining. Red pulp macrophages and Marginal zone macrophages were visualized by anti-F4/80(green, upper) and anti-CD169 (green, lower), respectively. The images are representative of at least 5 mice in each strain. **C:** Sera from 2 and 4 month old w.t. and MFG-E8-/- female mice were incubated with apoptotic cells. Binding intensities of IgG2c (MFI) to apoptotic cells were determined by flow cytometry. To investigate the effect of injecting exogenous apoptotic cells, 4 month old w.t. mice were injected with 20x 10^6^ apoptotic thymocytes every three days for 5 times and their serum samples were collected one week after the final injection. **D:** Levels of IgG2c antibody against MDA-LDL in the sera of 4 month old w.t. and MFG-E8-/- female mice were determined by ELISA. In A-D, each data point represents an individual animal.

**Fig 2:**
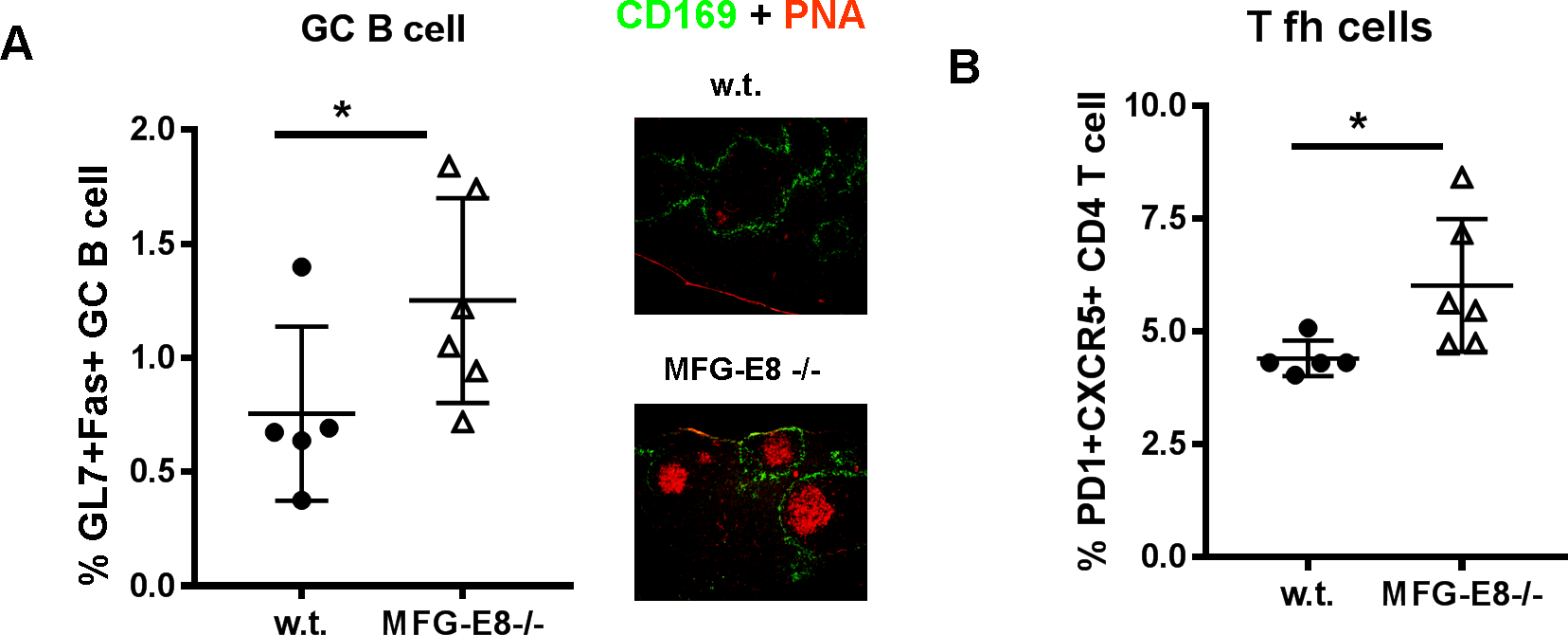
Increased percentages of GC B cell and T follicular helper cells in 4 month old MFG-E8-/- B6 mice. **A**: The percentages of GL7+Fas+ GC B cell in B220+ cells (**A left**) in the spleens of 4 month old w.t. and MFG-E8-/- female mice (left). Spleen sections were also stained with anti-CD169 and PNA to visualize GCs (right). The images are representative of at least 5 mice in each strain. **B:** The percentages of PD1+CXCR5+ T follicular helper cells in CD4+ cells in the spleens of 4 month old w.t. and MFG-E8-/- female mice. In A and B, each data point represents an individual animal.

**Fig 3:**
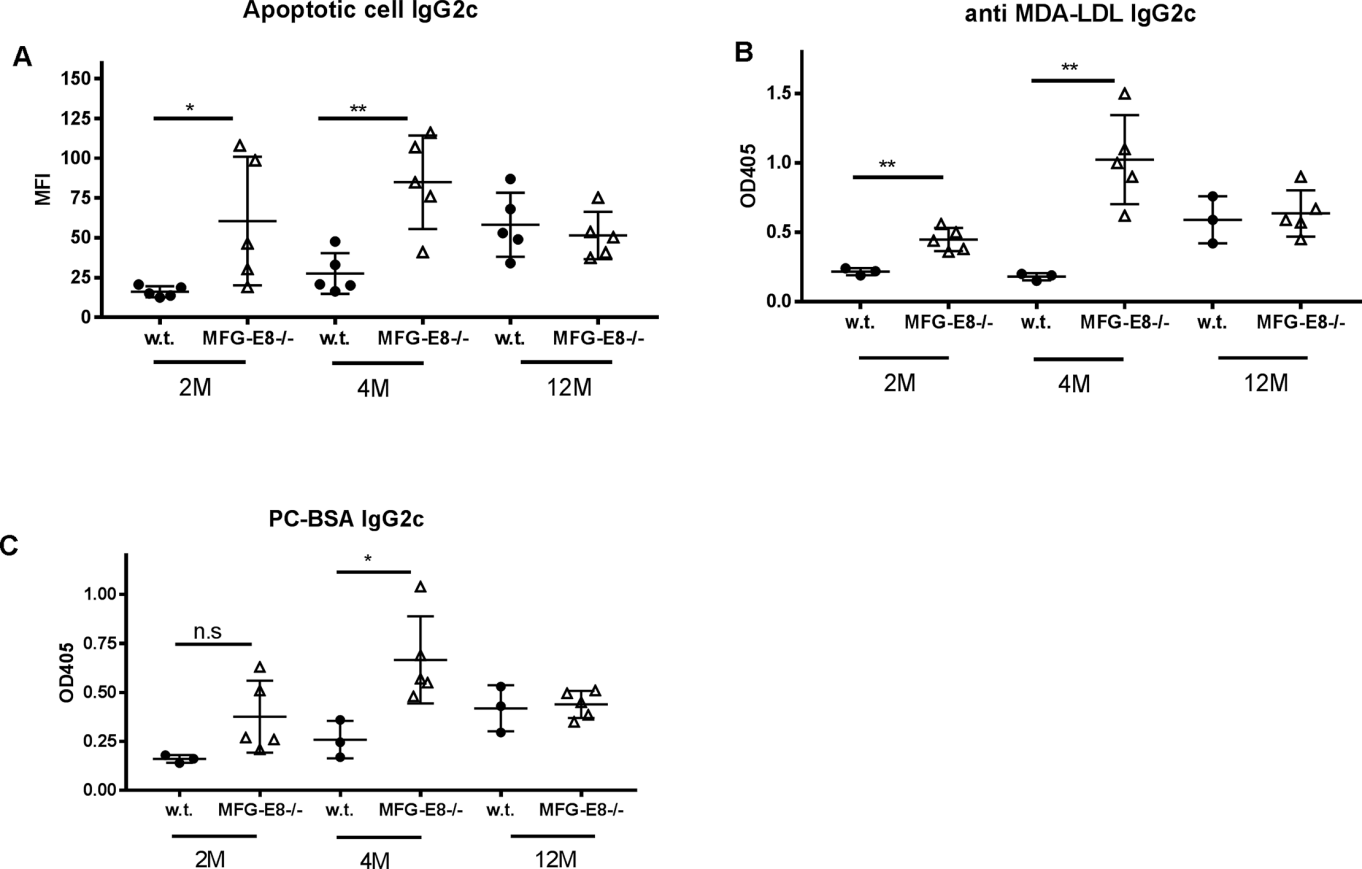
Time course of IgG2c response against apoptotic cell related antigens in MFG-E8-/- female mice. Serum samples were collected from w.t. and MFG-E8-/- mice of indicated ages. **A**) Serum levels of IgG2c against apoptotic cells were determined by flow cytometry. **B**) Serum levels of IgG2c against MDA-LDL were determined by ELISA. **C**) Serum levels of IgG2c against PC-BSA were determined by ELISA. Each data point represents an individual animal.

MFG-E8-/- mice in a B6 background did not develop overt autoimmunity [24, 25]. To determine whether accumulation of apoptotic cells in MFG-E8-/- B6 mice can induce some degree of humoral responses to self, we first evaluated antibody levels in these mice. As shown in **Fig 1A**, serum samples from MFG-E8-/- mice had elevated level of total IgG, which could be attributed to the increased levels of IgG2b and IgG2c subclasses. Immunofluorescence staining of the spleen sections using IgG2c specific antibody showed increased numbers of IgG2c+ plasma cells in both follicles and red pulp (**Fig 1B**). When serum samples from 4 month old MFG-E8-/- females were incubated with apoptotic cells, we found a significant increase of IgG2c binding to dying cells (**Fig 1C**), suggesting accumulation of apoptotic cells was able to induce spontaneous B cell responses, including class switching. On the other hand, repeated injection of exogenous apoptotic cells failed to induce anti-apoptotic cell IgG2c in 4 month old w.t. mice (**Fig 1C**). Since apoptotic cells share similar epitopes with oxidized LDL [26], we assessed the anti-MDA-LDL IgG2c levels in MFG-E8-/- mice. As shown in **Fig 1D**, sera from MFG-E8-/- mice had increased amounts of IgG2c against MDA-LDL. Consistent with the increased production of autoantibodies against apoptotic cells, 4 month old MFG-E8-/- female mice had increased numbers of GL7+Fas+ GC B cells and PD1+CXCR5+ T follicular helper cells (**Fig 2A and 2B**). The elevated antibody responses to various self antigens in MFG-E8-/- mice are most evident at 4 months of age and we no longer detected significant differences between w.t. and knock-out in mice older than 12 months (**Fig 3**). As old mice generally have less organized splenic architecture, and activated T cells accumulate in the spleens of old MFG-E8-/- mice, we mainly used 4 month old mice in subsequent studies to avoid the effects caused by these changes.

**Fig 4:**
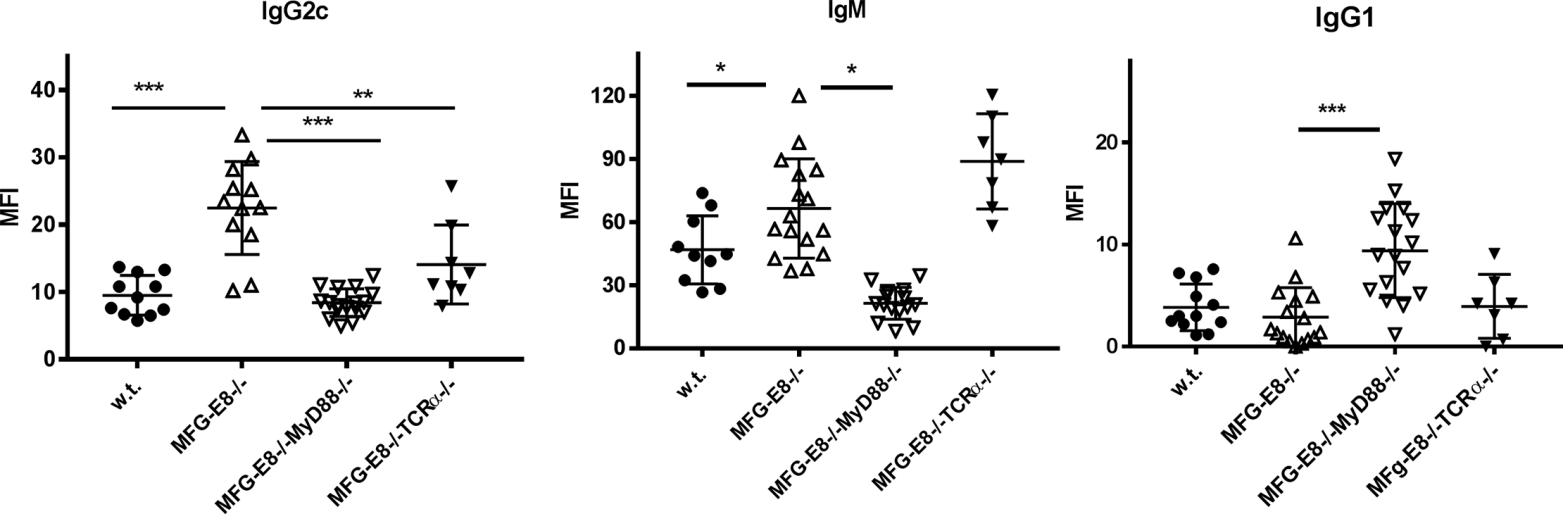
Anti-apoptotic cell IgG2c response in MFG-E8-/- mice depends on MyD88 and T cell help. Anti-apoptotic cell antibody binding from serum samples collected from 4 month old female mice of indicated genotypes. Each data point represents an individual animal.

The TLR-MyD88 pathway and T cell help are required to generate autoreactive IgG responses in autoimmune mice. Nucleic acids contained within immune complexes are known to provide endogenous TLR9 and TLR7 ligands to co-stimulate B cells. Although anti-dsDNA and anti-ssRNA antibodies are absent in MFG-E8-/- B6 mice, apoptotic cells may provide other TLR stimuli to B cells to facilitate autoantibody development [27]. To determine whether TLR signaling and T cell help are required for the antibody response against apoptotic cells, we crossed MFG-E8-/- mice with MyD88-/- mice and TCRα-/- mice. As shown in **Fig 4 (left panel)**, the IgG2c response to apoptotic cells in MFG-E8-/- mice was completely abrogated in MFG-E8-/-MyD88-/- mice. This response was also significantly reduced in MFG-E8-/-TCRα-/- mice. On the other hand, unlike IgG2c but similar to the response to foreign antigens, MyD88 deficiency increased the IgG1 response to apoptotic cells (**Fig 4 right panel**).

### Enhanced MZ B cell function in MFG-E8-/- B6 mice

**Fig 5:**
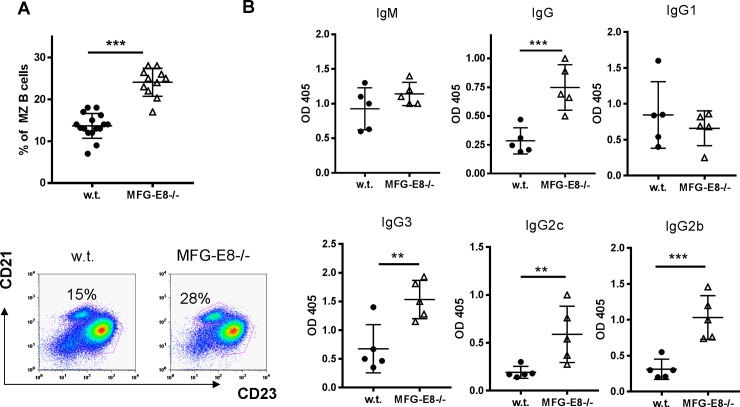
Enlarged MZ B cell compartment and enhanced antibody response to NP-Ficoll in MFG-E8-/- mice. **A:** The percentages of (CD19+/CD21high/CD23low) MZ B cells in the spleens of 4 month old w.t. and MFG-E8-/- female mice were determined by flow cytometry. Each data point represents an individual animal. **B**: Four month old w.t. and MFG-E8-/- female mice were challenged with NP-Ficoll. Serum titers of IgM and IgG (1–3) against NP were determined by ELISA at day 7 after the challenge. Each data point represents an individual animal. Similar results were obtained from male mice.

MZ B cells include many poly-reactive B cells that can be stimulated by self antigens released from apoptotic cells. Defective clearance of apoptotic cells in MertK-/- mice increased the percentage of MZ B cells [28]. Like Mertk-/- mice, we observed a significant increase of MZ B cells in MFG-E8-/- mice (**Fig 5A**). One important function of MZ B cells is to mediate the T cell independent antibody response. When challenged with the Type II T independent antigen NP-Ficoll, MFG-E8-/- mice produced significantly more anti-NP IgG, in particular IgG3, IgG2c, and IgG2b subclasses than w.t. mice (**Fig 5B**).

**Fig 6:**
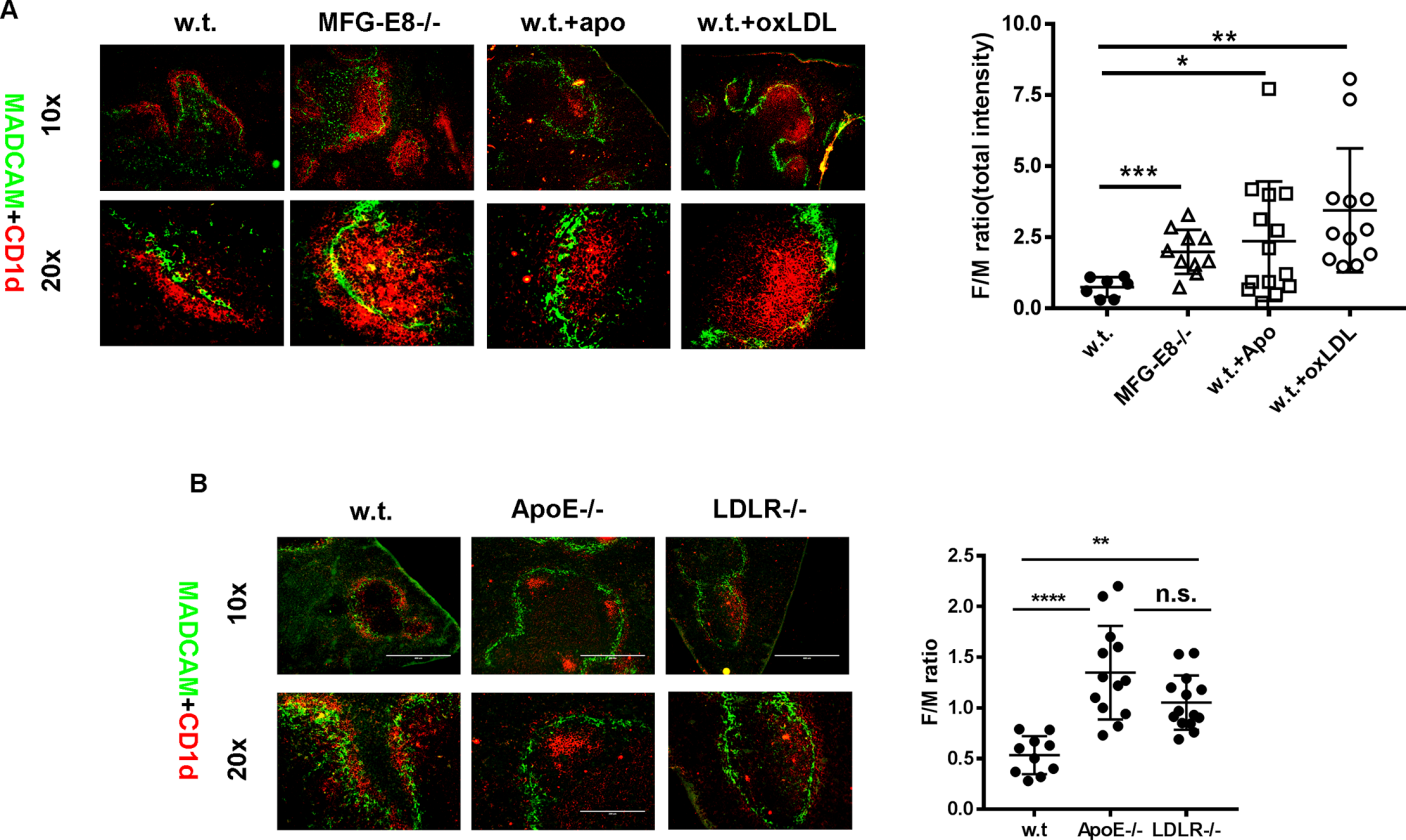
Translocation of MZ B cells in MFG-E8-/- mice. **A**: Left: Immunofluorescence staining of CD1d+ MZ B cells in the spleens of 10–12 month old w.t. and MFG-E8-/- female mice. To mimic the effect of apoptotic cell accumulation in MFG-E8-/- mice, 4–5 month old w.t. mice were injected with 20x 10^6^ apoptotic cells (apo) or 100μg oxidized LDL. The location of CD1d+ cells was examined 48 hours after injection. (Green–anti-MADCAM, red-anti-CD1d). Representative images are shown. Right: The ratios of CD1d+ MZ B cells within the follicle to those in the marginal zone (F/M ratio) were quantified by Image J. **B**: Left: To determine the effect of physiological accumulation of LDL, the location of CD1d+ cells in the spleens of 3 month old w.t., ApoE-/-, and LDLR-/- mice was evaluated. Representative images are shown. Right: The ratios of CD1d+ MZ B cells within the follicle to those in the marginal zone (F/M ratio) were quantified by Image J. In A and B, each data point represents the average of 5 follicles from an individual mouse.

Although the majority of MZ B cells are found in the marginal zone, they can shuttle between the MZ zone and follicle. After stimulation by TLR ligands, MZ B cells were released from the MZ zone and their translocation into the follicles was accelerated [29]. Since apoptotic cells may release endogenous TLR-like signals to promote MZ B cell migration, we examined the location of MZ B cells in 10–12 month old MFG-E8-/- mice. We stained spleen sections with anti-CD1d to locate MZ B cells. As shown in **Fig 6A**, whereas most CD1d+ MZ B cells remained in the marginal zone in old w.t. mice, a large fraction of them in age matched MFG-E8-/- mice were found in the follicle. In w.t. mice, the ratio of CD1d+ cells in the follicle to those in the MZ zone was about 0.75:1. On the contrary, in MFG-E8-/- mice, the same ratio was increased to 2:1.

To confirm that the translocation of MZ B cells in MFG-E8-/- mice is caused by the accumulation of apoptotic cells, we injected 3 month old w.t. mice with 20 x 10^6^ apoptotic cells. As shown in **Fig 6A,** after the injection of exogenous apoptotic cells, the ratios of follicular to MZ zone CD1d+ B cells were significantly increased in w.t. mice. One possible TLR ligand derived from apoptotic cells is the oxidized LDL mimetope [9, 30]. To determine whether oxidized LDL can directly induce MZ B cell translocation, we injected w.t. mice with 100μg oxidized LDL. As shown in **Fig 6A**, similar to exogenous apoptotic cells, exogenous oxidized LDL significantly increased the follicular/MZ CD1d+ B cell ratio from 0.75 to 3.5. To further demonstrate the effect of LDL on MZ B cells, we asked whether a physiological increase of LDL could also induce MZ B cell translocation. To this end, we examined the location of CD1d+ MZ B cells in 3–5 month old ApoE-/- and LDLR-/- mice [30]. As shown in **Fig 6B**, the mean ratio of follicular CD1d+ cells to MZ CD1d+ cells was increased from 0.5 in w.t. mice to 1.5 in age matched ApoE-/- mice. LDL mediated MZ B cell translocation was only partially mediated by the LDL receptor, as LDLR deficiency only caused a modest reduction of CD1d+ MZ B cells in the follicles (**Fig 6B**).

**Fig 7:**
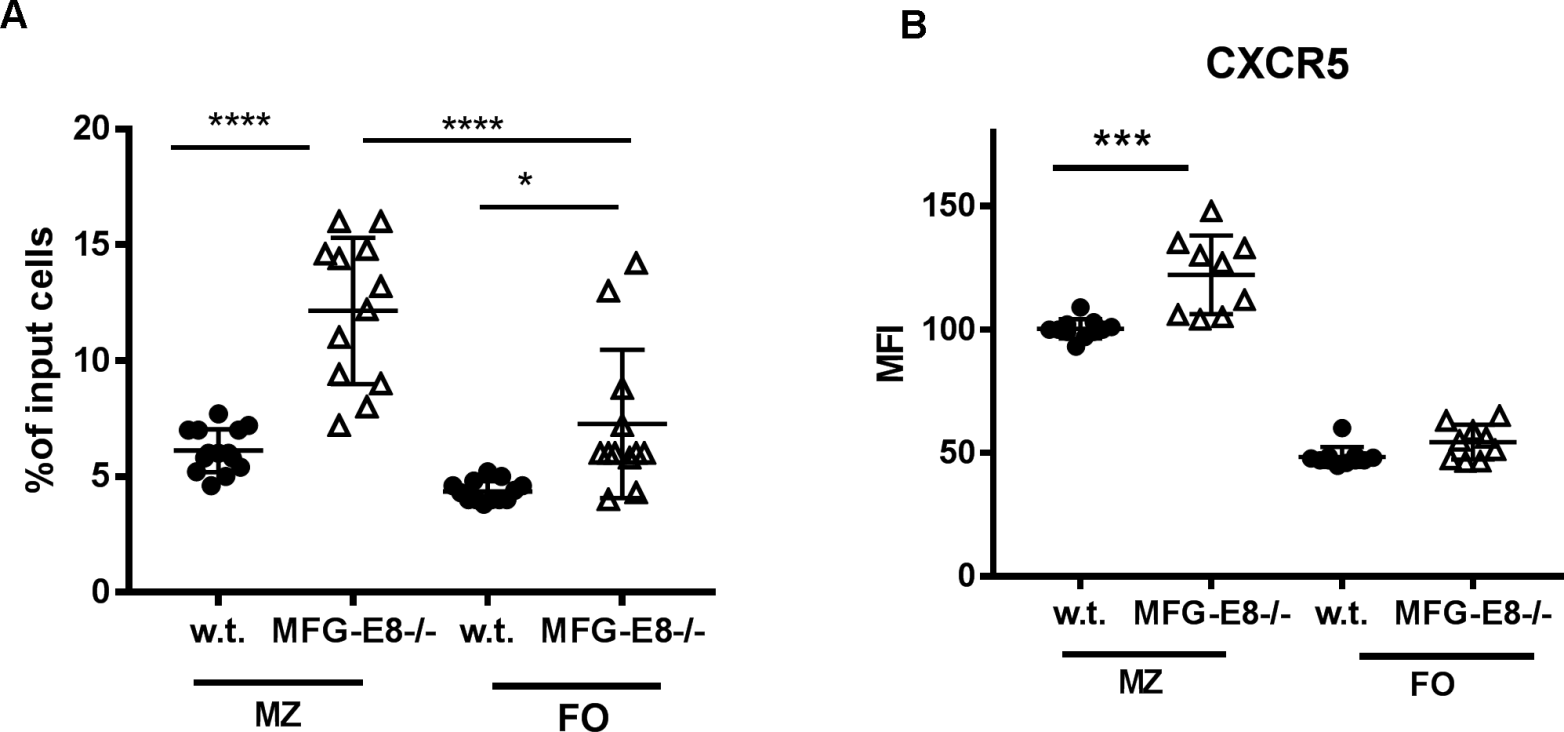
MZ B cells from MFG-E8-/- mice exhibit enhanced migratory response to CXCL13 and increased expression of CXCR5. **A**: Trans-well migration assay was used to determine the migratory responses to CXCL13 by splenic B cells from 4 month old w.t. and MFG-E8-/- mice. MZ- MZ zone B cells, FO- follicular B cells. The phenotype and the number of migrated cells were determined by flow cytometry. The percent of migrated cells = number of cells in the bottom well/number of input cells. **B:** The expression levels of CXCR5 on the surface of MZ and FO B cells in 4 month old w.t. and MFG-E8-/- female mice. In A and B, each data point represents an individual animal.

Since the migration of B cells into the follicle is mainly mediated through chemokine CXCL13 and its receptor CXCR5 [6], we next asked whether the increased presence of MZ B cells in the follicles of MFG-E8-/- mice is associated with enhanced expression of CXCR5 and increased migratory response to CXCL13. To this end, we used a transwell assay to evaluate how w.t. and MFG-E8-/- B cells respond to CXCL13 *in vitro*. As shown in **Fig 7A**, splenic B cells from MFG-E8-/- mice, in particular MZ B cells are more responsive to CXCL13 induced trans-migration. These B cells also expressed higher levels of CXCR5 on their surface than w.t. MZ B cells (**Fig 7B**).

### MFG-E8 deficiency promotes MZ B cell differentiation of anti-dsDNA 56R/Vκ38c B cells

**Fig 8:**
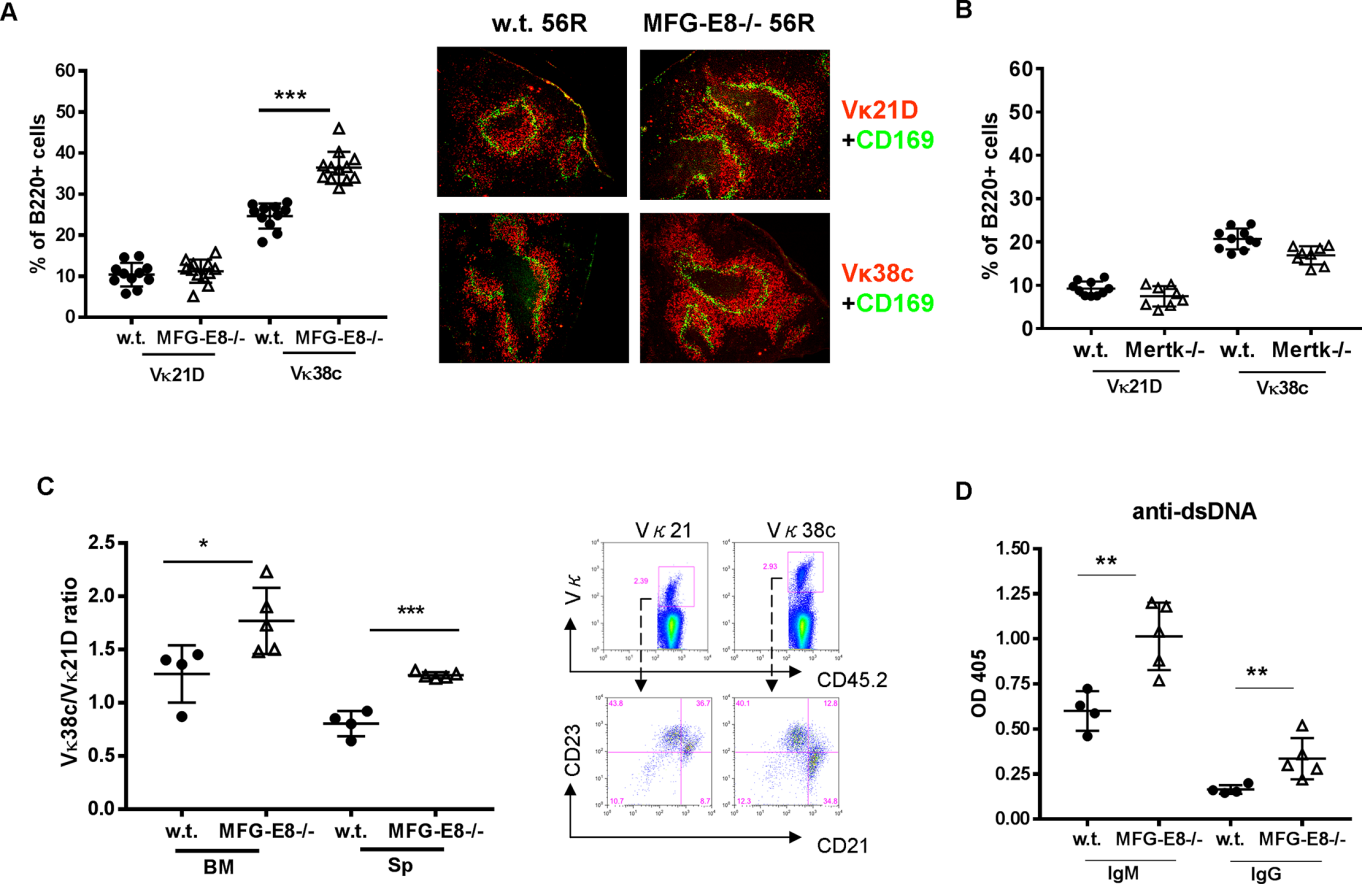
MFG-E8 deficiency selectively promotes MZ B cell differentiation of anti-dsDNA specific 56R/Vκ38c B cells. **A:** MFG-E8-/- mice were crossed with 56R anti-dsDNA BCR transgenic mice. The percentages of non-autoreactive 56R/Vκ21D and autoreactive 56R/Vκ38c B cells in the spleens of 3 month old female mice were determined by idiotype specific antibodies using flow cytometry (left) and Immunofluorescence staining (right). The images are representative of at least 5 mice in each strain. **B**: Mertk-/- mice were crossed with 56R mice. The percentages of non-autoreactive 56R/Vκ21D and autoreactive 56R/Vκ38c in the spleens were determined by flow cytometry. **C**: Bone marrow from CD45.1+ w.t. or MFG-E8-/- mice was mixed with genotype matched bone marrow from CD45.2+56R mice at a 1:1 ratio. The mixed bone marrow cells were used to reconstitute irradiated CD45.1+ w.t. or MFG-E8-/- mice. At 3–4 month after the reconstitution, spleen (SP) and bone marrow (BM) cells derived from 56R mice were distinguished by the CD45.2 congenic marker. The ratios of autoreactive 56R/Vκ38c to non-autoreactive 56R/Vκ21D (left) and their surface phenotypes (right) were determined by flow cytometry. **D**: Serum titers of anti-dsDNA IgM and IgG from the bone marrow chimeras constructed in C were determined by ELISA. In C and D, the results are representative of two independent experiments. In A-D, each data point represents an individual animal.

In addition to oxidized LDL, apoptotic cells also release blebs containing DNA and RNA. Although in the non-autoimmune B6 background, MFG-E8 deficiency did not break central tolerance and old MFG-E8-/- B6 mice did not develop anti-dsDNA and ssRNA antibodies, nucleic acids from apoptotic cells can provide both self antigens and TLR signals in the periphery to stimulate autoreactive B cells that have escaped bone marrow selection. To determine how apoptotic cells in MFG-E8-/- mice promote the differentiation of autoreactive anti-dsDNA B cells in the periphery, we crossed MFG-E8-/- with 56R transgenic mice whose production of anti-dsDNA antibody was shown to be TLR9 dependent [31]. 56R BCR transgenic mice express a knock-in heavy chain that is specific to dsDNA. The forced expression of BCR allows them to escape from central deletion in bone marrow [32]. The specificity of 56R heavy chain can be modified by light chains. Combination with Vκ38c retained 56R’s reactivity to self, whereas combination with the Vκ21D light chain eliminates its auto-reactivity. [21]. In this study, we used idiotype specific antibodies to distinguish non-autoreactive 56R/Vκ21D from auto-reactive 56R/Vκ38c B cells [21]. As shown in **Fig 8A**, compared to w.t. 56R mice, autoreactive 56R/Vκ38c but not non-autoreactive 56R/Vκ21D B cells were significantly increased in the spleens of MFG-E8-/-56R mice. Immunofluorescence staining of the spleen sections revealed that most of the expansion of 56R/Vκ38c B cells occurred within the MZ. However, unlike the polyclonal MZ B cells in old MFG-E8-/- B6 mice, most of 56R/Vκ38c B cells in MFG-E8-/- 56R mice remained outside of the follicles, suggesting dsDNA is unlikely to be the ligand that induced MZ B cells to translocate. Interestingly, although Mertk deficiency promoted the expansion of total MZ B cells, we did not observe any increase of 56R/Vκ38c B cells in Mertk-/- 56R transgenic mice (**Fig 8B**).

Since the dominance of transgenic B cells can skew the selection of B cells [33], we wanted to examine the effect of MFG-E8 deficiency in a more physiologically poly-clonal condition. We mixed bone marrow cells from w.t. and 56R transgenic mice and used them to reconstitute irradiated hosts. We then used the ratio of 56R/Vκ38c to 56R/Vκ21D cells to assess their selection in respective hosts. As shown in **Fig 8C**, in both bone marrow and spleen, the mean ratio of 56R/Vκ38c to 56R/Vκ21D was significantly higher in MFG-E8-/- hosts than in w.t. hosts. Moreover, greater than 50% of 56R/Vκ38c B cells had differentiated into CD21high/ CD23low MZ B cells, whereas most of non-autoreactive 56R/Vκ21D B cells were either CD21low/CD23high follicle B cells or CD21high/CD23high T2 transitional B cells [21, 34] (**Fig 8C right panel**). In agreement with the preferential selection of autoreactive B cells, MFG-E8-/- BMCs (bone marrow chimeras) had higher levels of anti-dsDNA antibody than w.t. BMCs (**Fig 8D**). However, despite the expansion of 56R/Vκ38c B cells, GC B cells and antibody producing AFCs (antibody forming cells) were absent in MFG-E8-/- 56R mice, nor did we detect any significant deposition of autoantibody in the kidneys. These observations suggest that although TLR 9 signaling derived from apoptotic cell DNA can promote the positive selection of anti-dsDNA B cells, it is not sufficient to drive them to differentiate into AFCs.

### MFG-E8 deficiency promotes the differentiation of anti-ssRNA B cells

**Fig 9:**
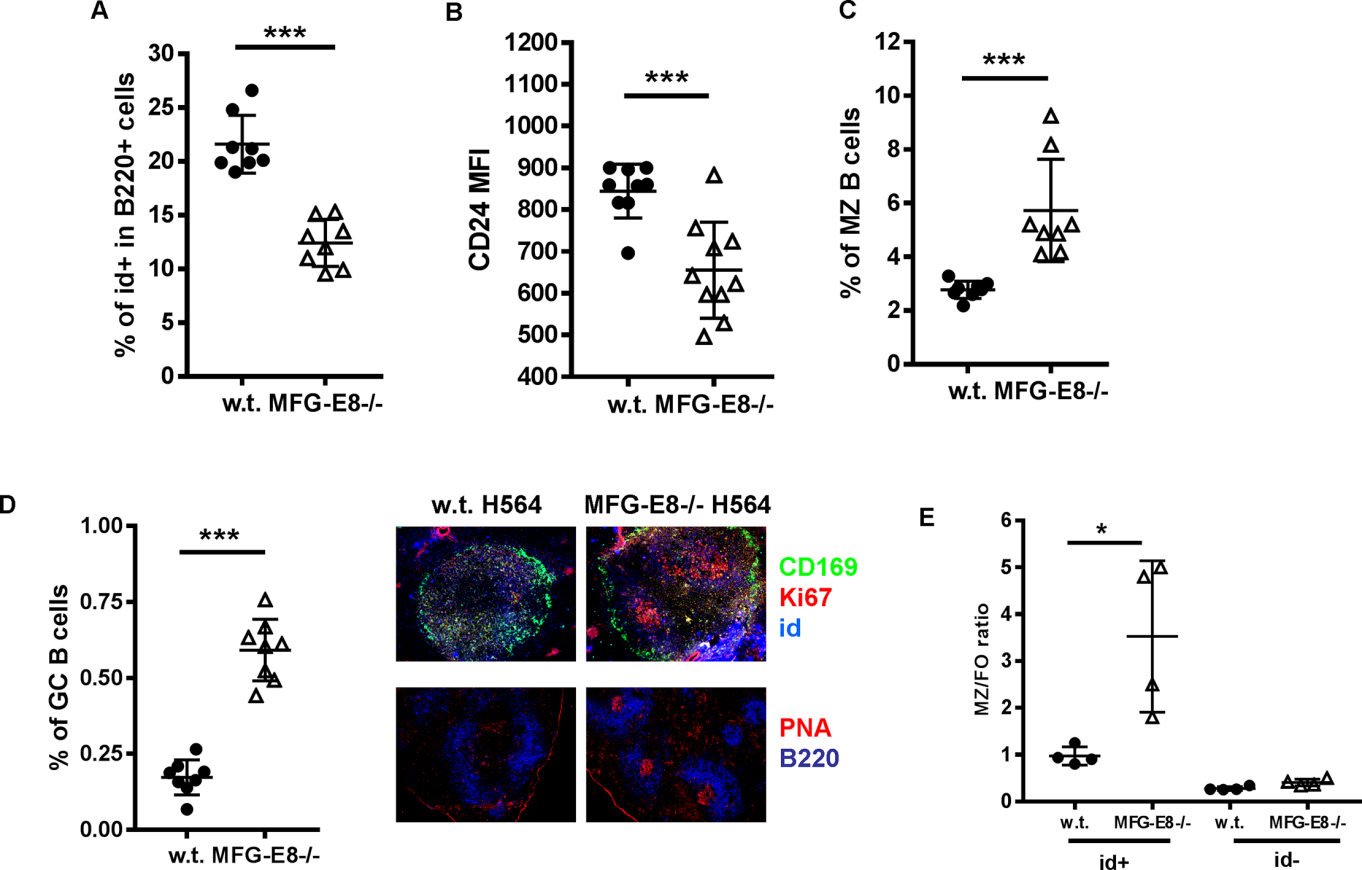
MFG-E8 deficiency promotes the differentiation of anti-ssRNA specific H564 B cells. **A-D**: MFG-E8-/- mice were crossed with H564 anti-ssRNA BCR transgenic mice. The percentages and surface phenotypes of id+ B cells in 3 month old w.t. and MFG-E8-/- H564 mice were determined by flow cytometry using an idiotype specific antibody. A) The percentages of id+ H564 B cells in the spleens. B) Surface expression of CD24 on id+ B cells. C) The percentages of CD21high/CD23low id+ MZ B cells. D) Left: the percentages of Fas+GL7+ id+ GC B cells, right: immunofluorescence staining of the spleen sections from w.t. and MFG-E8-/- H564 mice. Top: CD169 (green), Ki67 (red), id (blue). Note that large numbers of id+ plasma cells (blue) were detected around CD169+ macrophages in MFG-E8-/-H564 mice. Bottom: PNA (red), B220 (blue). The images are representative of at least 5 mice in each strain. **E:** Spleen cells from CD45.2+ H564 BCR transgenic mice were transferred into w.t. and MFG-E8-/- hosts (CD45.1+). Five days later, Id+ H564 and id- cells in the spleens of the recipients were detected by flow cytometry and the ratios of marginal zone B cells to follicular B cells were calculated. The result is representative of two independent experiments. In A-E, each data point represents an individual animal.

In addition to DNA, defective clearance of apoptotic cells can also release cytoplasmic RNA to engage TRL7 on B cells. The dichotomy of TLR9 and TLR7 pathways in lupus suggests anti-dsDNA B cells and anti-ssRNA B cells may respond differently to their respective autoantigens. To determine how MFG-E8 deficiency drives the differentiation of ssRNA specific autoreactive B cells in the periphery, we crossed MFG-E8-/- mice with H564 BCR transgenic mice. The production of autoantibodies in H564 mice depends on TLR7 signaling [22]. In H564 BCR mice, both heavy and light chains were replaced by the respective chains from the autoreactive B cell clone [22]. SSRNA specific B cells in H564 mice can be detected by an idiotype specific antibody [22]. Consistent with the published results, in w.t. H564 mice, 20% of B cells are id+ (idiotype positive) [22]. Surprisingly, MFG-E8 deficiency significantly reduced the total percentages of id+ B cells in H564 mice (**Fig 9A**). On the other hand, the percentages of id+ MZ and id+ GC B cells were increased in MFG-E8-/-H564 mice (**Fig 9C and 9D**), suggesting MFG-E8 deficiency might accelerate the differentiation of H564 B cells. The increase of GC B cells in MFG-E8-/- mice was confirmed by immunofluorescence staining of the spleen sections with PNA as well as the proliferation marker Ki67 (**Fig 9D right panel**). Coincidentally with their accelerated differentiation, immature B cell marker, CD24, on id+ B cells was substantially down regulated on MFG-E8-/-H564 B cells (**Fig 9B**). To further demonstrate the effect of MFG-E8 deficiency in a poly-clonal condition, we directly transferred spleen cells from H564 BCR mice into w.t. and MFG-E8-/- host. Seven days later, we evaluated the phenotype of id+ B cells in the recipients. In MFG-E8-/- hosts, we found the ratio of MZ to FO B cells was significantly increased in id+ but not id- B cells (**Fig 9E**).

**Fig 10:**
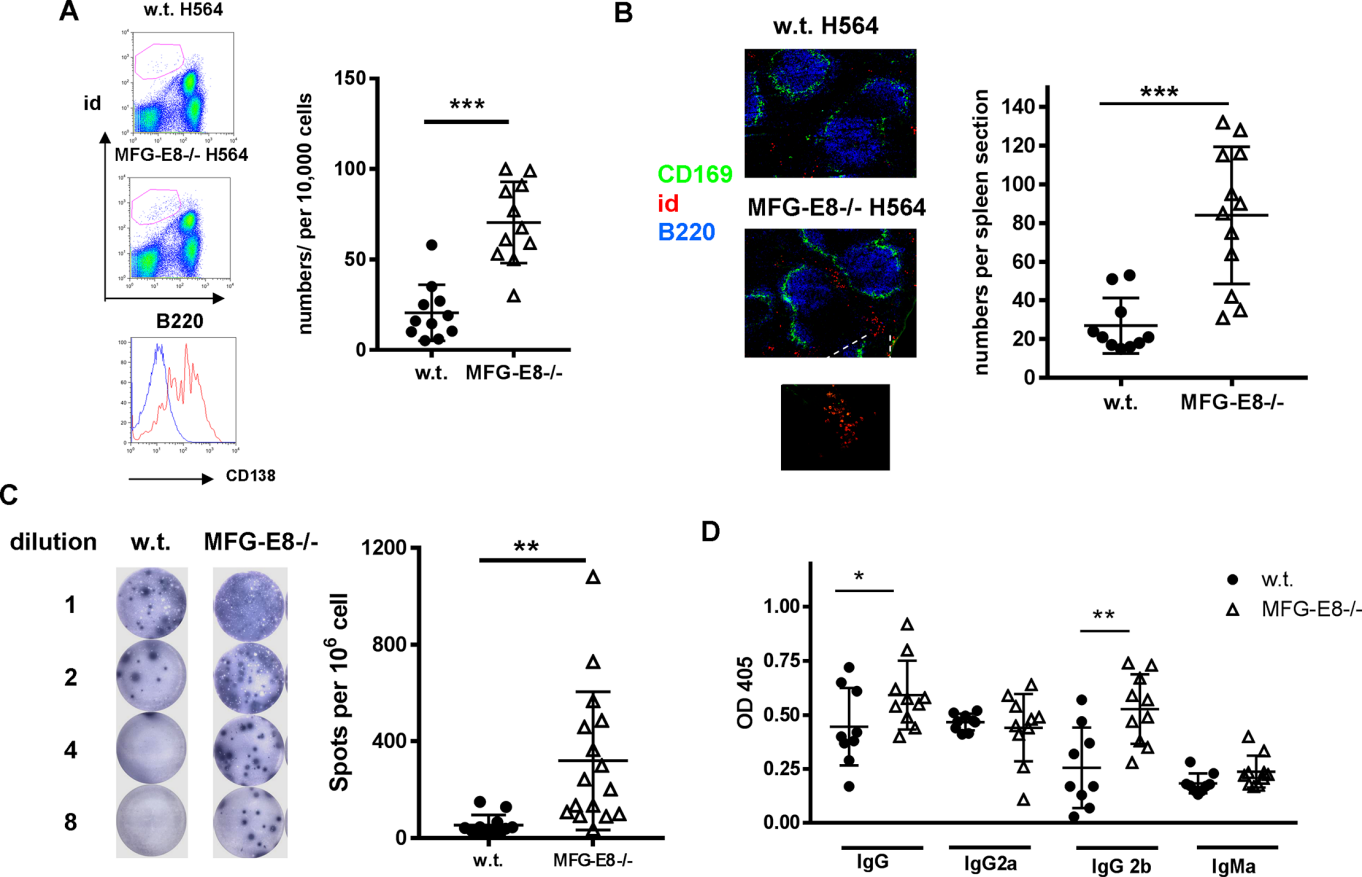
MFG-E8 deficiency promotes plasma B cell differentiation of anti-ssRNA specific H564 B cells. Plasma cell differentiation of id+ B cells was examined in the spleens of 3 month old w.t. and MFG-E8-/- H564 BCR transgenic mice. **A:** The numbers of id+/B220low plasma cells per 10,000 id+ cells in the spleens were enumerated by flow cytometry. Surface expression of CD138 on id+/B220low cells was significantly higher than id+/B220+ cells. (Histogram: blue—id+/B220+high, red—id+/B220low). **B:** To locate id+ plasma cells in the spleens, frozen spleen sections from w.t. and MFG-E8-/- H564 mice were stained with anti-CD169(green), anti-B220(blue), and idiotype specific antibody (red). Right: The number of id+ plasma cells in each spleen section was enumerated using Image J. Each data point represents the average of three sections from an individual animal. **C:** The numbers of id specific IgG producing H564 B cells in the spleens of w.t. and MFG-E8-/-BCR transgenic mice were determined by ELISPOT. **D**: The titers of id specific IgG, IgG2a, and IgG2b antibodies in the sera of 4 month old w.t. and MFG-E8-/-H564 mice were determined by ELISA. In A-D, each data point represents an individual animal.

**Fig 11:**
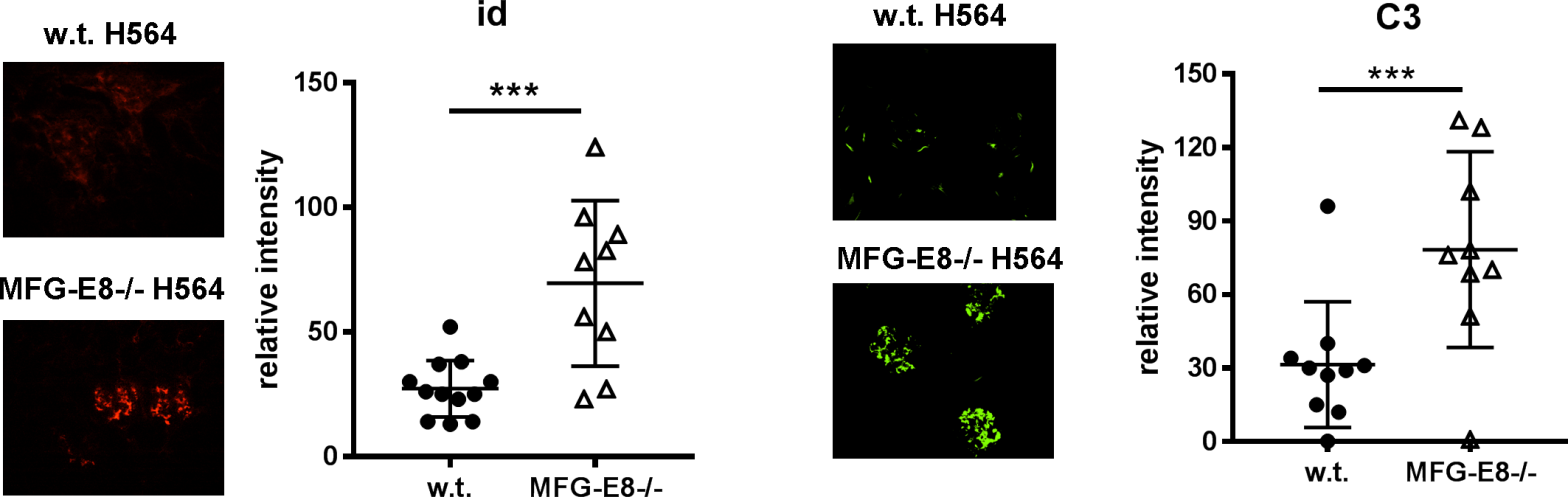
Increased depositions of anti-ssRNA antibody and C3 in the kidneys of MFG-E8-/-H564 mice. To detect depositions of anti-ssRNA and C3, kidney sections from 4 month old w.t. and MFG-E8-/- H564 mice were stained with antibody specific for id (left) and C3 (right). The staining intensities in the glomeruli were quantified by Image J. Each data point represents the average of 5 glomeruli from an individual animal.

The reduction of id+ H564 B cells in MFG-E8-/- mice prompted us to investigate whether these cells had differentiated into autoantibody producing plasma cells (AFCs). B cell receptor and other typical B cell markers such as B220 and CD19 are down-regulated in plasma cells. In w.t. H564 mice, we found a small percentage of id+ B cells expressing low levels of B220 as well as high levels of the plasma cell marker CD138, suggesting they were autoantibody producing plasma B cells. In the absence of MFG-E8, the numbers of id+, B220low/CD138high plasma cells were significantly increased (**Fig 10A**). The increase in autoantibody producing B cells was confirmed by immunofluorescence staining of the spleen sections using an idiotype specific antibody. As shown in **Fig 10B**, most of the autoantibody producing id+ cells in MFG-E8-/- mice were in the red pulp. We also confirmed the increase of IgG producing plasma B cells in MFG-E8-/- H564 mice by ELISPOT assay. As shown in **Fig 10C**, the mean number of id specific IgG producing cells was increased from 55/ 10^6^ in w.t. mice to 320/10^6^ in MFG-E8-/- mice. Consistent with the increased number of plasma cells, the level of id specific IgG, in particular the IgG2b subclass, was substantially higher in MFG-E8-/-H564 mice than in w.t. H564 mice (**Fig 10D**). In addition, we detected a significantly higher amount of id specific autoantibody and C3 deposited in the kidneys of MFG-E8-/-H564 mice than in w.t. H564 mice (**Fig 11**).

### Activated 56R B cells and H564 B cells react differently to apoptotic cell accumulation in MFG-E8-/- mice

**Fig 12:**
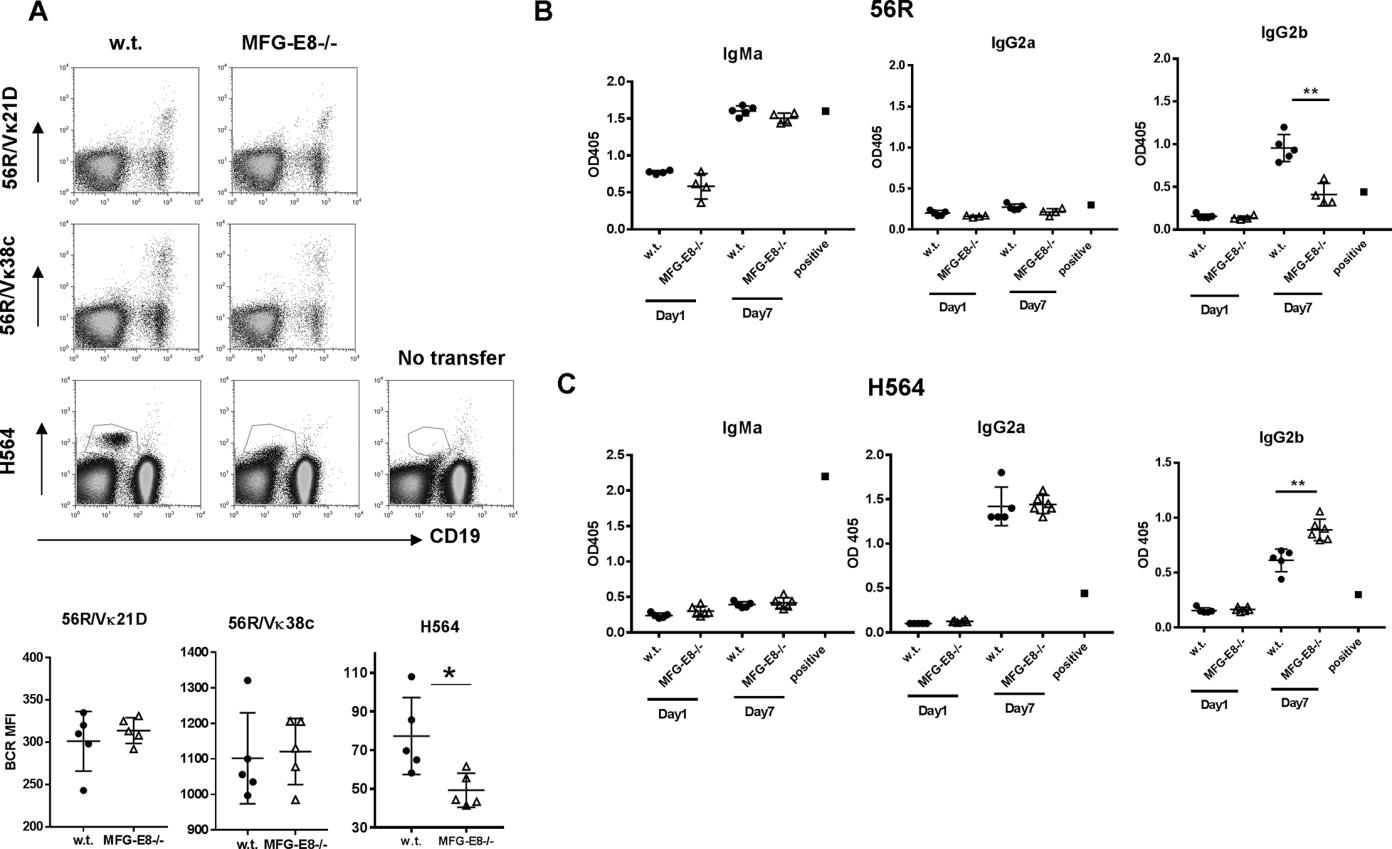
Activated 56R and H564 B cells show different sensitivities to apoptotic cell accumulation in MFG-E8-/- mice. **A:** CD45.2+ 56R and H564 B cells were activated by LPS (1μg/ml) for 3 days. Activated B cells were extensively washed and transferred into w.t. and MFG-E8-/- hosts (CD45.1+). At day 5 after the transfer, id+ autoreactive B cells in the circulation were identified by idiotype specific antibodies. The surface levels of BCR on transferred cells were determined by flow cytometry. The expression levels of various BCRs in w.t. and MFG-E8-/- recipients were summarized below. The results are representative of two experiments. **B:** Activated 56R B cells were transferred as in A. At day 1 and day 7 after the transfer, sera were collected from the recipients. The levels of autoantibody produced by transferred 56R/Vk38c B cells in w.t. and MFG-E8-/- recipients were determined by ELISA using an idiotype specific antibody. Allotype specific anti-IgMa and anti-IgG2a were used to distinguish them from the endogenous IgMb and IgG2c in the recipients. The results are representative of three experiments. Supernatant from *in vitro* LPS stimulated 56R B cells was used as a positive control. The control contained approximately 1μg/ml antigen specific antibody. **C:** Activated H564 B cells were transferred as in B. The levels of autoantibody produced by transferred H564 B cells in w.t. and MFG-E8-/-recipients were determined by ELISA using idiotype specific antibody. Allotype specific anti-IgMa and anti-IgG2a were used to distinguish them from the endogenous IgMb and IgG2c in the recipients. The results are representative of two experiments. Supernatant from *in vitro* LPS stimulated H564 B cells was used as a positive control. The control contained approximately 1μg/ml antigen specific antibody. In B and C, note that neither 56R nor H564 B cells underwent class switching after 3 day LPS treatment *in vitro*.

**Fig 13:**
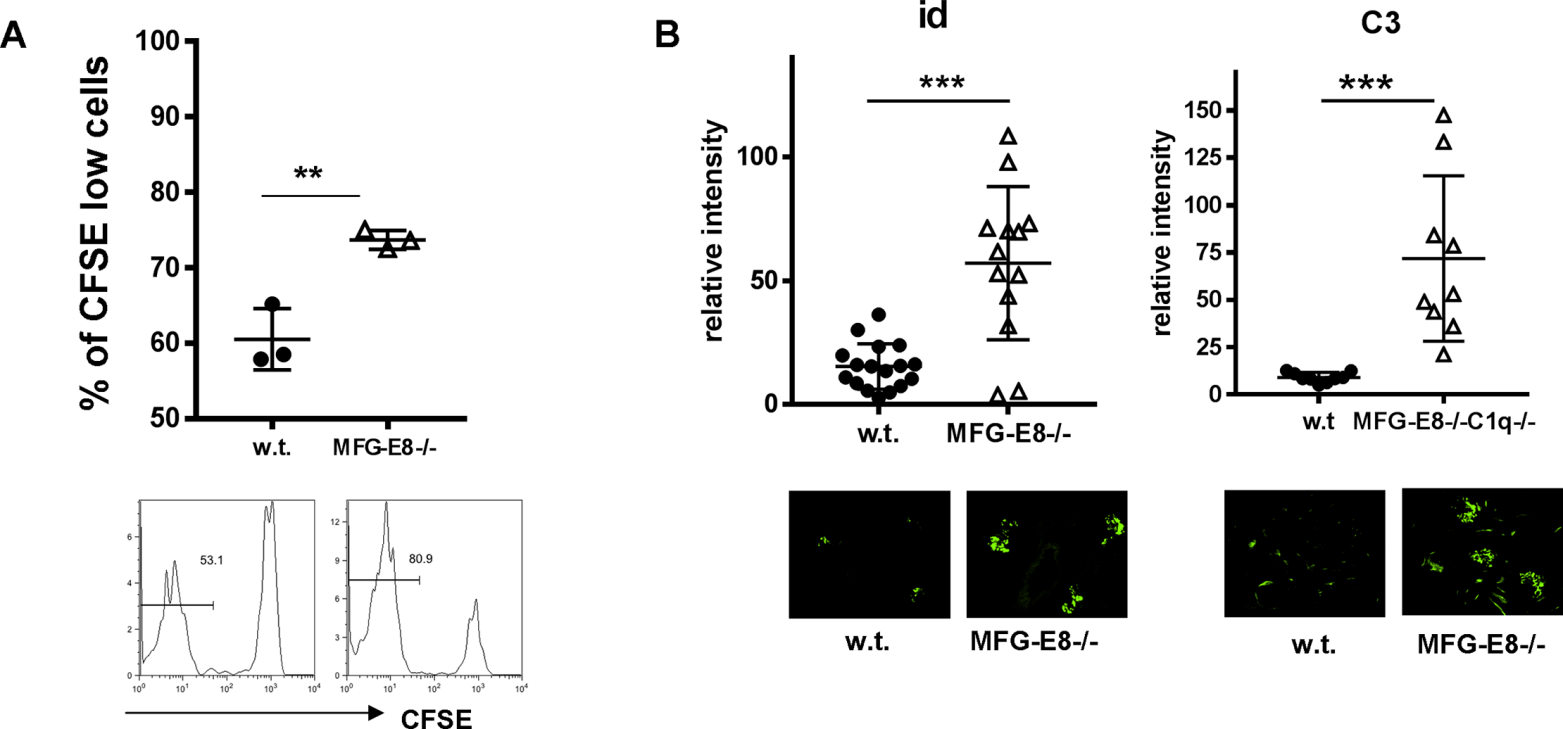
Increased proliferation of activated H564 B cells and depositions of anti-ssRNA antibody and C3 in the kidneys of MFG-E8-/- mice. **A:** LPS activated H564 B cells were labeled with CFSE before transferring into w.t. and MFG-E8-/- hosts. Three days later, the proliferation of transferred cells in the spleen was quantified by CFSE dilution. The result is representative of two experiments. **B:** Two weeks after the transfer of activated H564 B cells, depositions of auto-antibody and C3 in the recipients’ kidney were determined by immunofluorescence staining using antibodies specific for the idiotype and C3, respectively. The staining intensities were quantified by Image J. Each data point represents the average of 5 glomeruli from an individual animal.

To demonstrate the direct effect of excessive apoptotic cells on plasma cell differentiation in the periphery, we transferred activated autoreactive B cells into w.t. and MFG-E8-/-mice. We first activated 56R and H564 B cells with a low dose of LPS for 3 days. Both 56R and H564 B cells stimulated by this method remained IgMa+ before the transfer. After extensive washes, we transferred these activated B cells into w.t. and MFG-E8-/- hosts. At day 5 after the transfer, we evaluated the phenotype of id+ cells in the circulation. As shown in **Fig 12A**, id+ H564 B cells in MFG-E8-/- recipients had significantly lower levels of BCR on their surface, suggesting enhanced B cell activation and plasma cell differentiation. In contrast, MFG-E8 deficiency did not have a similar effect on activated autoreactive 56R/Vκ38c B cells. Using idiotype specific antibodies, we also determined the level of autoantibodies produced by the transferred cells. As shown in **Fig 12B and 12C**, consistent with their phenotypical changes, transferred 56R B cells produced significant amounts of IgMa and IgG2b that were idiotype specific, whereas transferred H564 B cells produced only IgG2a and IgG2b but not IgM that were idiotype specific. Most importantly, whereas MFG-E8 deficiency enhanced the IgG2b production by H564 B cells, it reduced the IgG2b production by 56R B cells (**Fig 12B and 12C)**. To evaluate the proliferation of activated H564 B cells *in vivo*, we labeled LPS activated H564 B cells with CFSE before the transfer. At day 5 after the transfer, significantly higher numbers of id+ splenic B cells had lost their CFSE staining in MFG-E8-/- recipients than in w.t. recipients (**Fig 13A**). Consistent with the enhanced differentiation of plasma cells, MFG-E8-/- recipients also had significantly more id+ IgG and C3 depositions in their kidneys than in w.t. recipients (**Fig 13B**).

## Discussion

In our study, we found MFG-E8 deficient mice had higher levels of IgG2b and 2c antibodies against apoptotic cells and oxidized LDL than w.t. mice. The class switching we observed is different than reported by Chikazawa et al., who described an increase of anti- IgM but not IgG against peroxidized lipids in MFG-E8-/- mice [35]. Although the reason for this discrepancy is not clear, our results demonstrated excess apoptotic cells, without any adjuvant, can induce a T cell-dependent response. Since TCRα deficiency only partially reduced the anti-apoptotic cell IgG2c response, an innate B cell population such as MZ B cells may also contribute to this response. In fact, MZ B cells are capable of producing anti-viral particle IgG antibodies without T cell help [36]. Surprisingly, we found both IgM and IgG2c responses against apoptotic cells were completely abrogated by MyD88 deficiency (**Fig 4**). Previous studies have firmly linked anti-dsDNA and anti-ssRNA antibody responses in murine lupus models to the TLR-MyD88 pathway [13, 14]. DsDNA and ssRNA together with their binding antibodies can engage TLR9 and TLR7 to provide essential co-stimulatory signals to perpetuate an autoreactive B cell reaction. However, antibodies against nucleic acids are absent in MFG-E8-/- mice in a B6 background. Therefore, apoptotic cells might provide other TLR ligands, such as HMGB-1 and oxidized LDL [37, 38], to co-stimulate the antibody response. As the B cell response to apoptotic cells is the precursor of autoantibody development, our results suggest the importance of the TLR-MyD88 pathway in autoantibody production is not limited to nucleic acid specific antibodies. The predominance of IgG2b and 2c subclasses in the autoantibody profile of 4 month old MFG-E8-/- mice differs from the IgM autoantibody induced by repeated injection of apoptotic cells [20, 39, 40]. Deposition of apoptotic cell fragments on FDCs in MFG-E8-/- mice may be required for class switching [18]. It is interesting to note that the autoantibody responses triggered by apoptotic cells were not sustained in old MFG-E8-/- mice (**Fig 3**). The strength of costimulatory signal(s) provided by non-nucleic acid stimuli might be weaker than those provided by nucleic acids.

Both MFG-E8-/- mice and other strains deficient in apoptotic cell removal have an expanded MZ B cell population. Due to their anatomical location, various poly-reactive B cell clones in the MZ can be positively selected by different self antigens released by apoptotic cells in circulation. In fact, both anti-dsDNA 56R/Vκ38c and anti-ssRNA H564 B cells were induced to differentiate into MZ B cells in MFG-E8-/- mice. The positive selection of these cells is likely to occur at the immature T2 B cell stage within the follicles, as we found in MFG-E8-/- 56R bone marrow chimera mice, in which, autoreactive 56R/Vκ38c B cells differentiated into FO and MZ B cells, whereas a significant percentage of non-autoreactive 56R/Vκ21D B cells retained the CD21high/ CD23high T2 B cell phenotype [34] (**Fig 8C**). The coincidence of the follicular location of T2 cells [34] and the accumulation of apoptotic cells at the same anatomical site in MFG-E8-/- mice [18] supports this hypothesis.

MZ B cells migrated into the follicles in old MFG-E8-/- mice. Apoptotic cells can release endogenous TLR ligands to trigger this response. Since injection of exogenous oxidized LDL and physiological accumulation of LDL in ApoE-/- and LDLR-/- mice induced a similar translocation, oxidized LDL mimetopes on the surface of apoptotic cells could be one of these ligands. The effect of oxidized LDL on MZ B cells is supported by a recent study. In that study, Grasset et al. demonstrated repeated injection of apoptotic cells or oxidized LDL, as well as ApoE deficiency, increased the percentage of MZ B cells [41]. Although the location of MZ B cells was not determined in the study, it is conceivable that oxidized LDL could have other impacts on MZ B cells in addition to promoting their expansion. Oxidized LDL released by apoptotic cells can drive MZ B cell translocation through both direct and indirect means. Grasset et al. also demonstrated direct binding of oxidized LDL to MZ B cells. Like other TLR ligands, oxidized LDL could directly induce the translocation of MZ B cells into follicles by activating the TLR4 pathway [38]. Since the MZ B cell migration was partially impaired in LDLR-/- mice, LDL receptor only plays a modest role in this reaction. Alternatively, oxidized LDL can trigger responses from macrophages in the marginal zone. Guillen et al. has shown that deletion of the cholesterol sensor, LXRa, led to marginal zone macrophage deficiency and consequent MZ B cell translocation [42]. How marginal zone macrophages respond to modified lipid is currently unknown. It is possible for activated macrophages to alter their interaction with MZ B cells. Similar to MFG-E8 deficient mice, injection of apoptotic cells or oxidized LDL increased the GC reaction [41]. The numbers of GC B cells were also increased in ApoE-/- mice. Translocation of MZ B cells into follicles may therefore facilitate the transportation of circulating antigens onto FDCs to trigger anti-self GC reactions. Since anti-dsDNA 56R/Vκ38c B cells were largely confined within the marginal zone in MFG-E8-/- 56R mice (**Fig 8A**), dsDNA is unlikely to be the ligand that induces MZ B cell migration into the follicles. Since increased antibody responses to oxidized LDL and MZ B cell migration into follicles have been reported in lupus patients, our observations hereby identified a missing link between these two phenomena. Since other stimuli such as IFN-α, can also induce MZ B cell translocation [43], we cannot exclude their involvement in apoptotic cell induced MZ B cell migration.

In MFG-E8-/- mice, ssRNA specific id+ H564 B cells differentiated into AFCs, whereas dsDNA specific 56R/Vκ38c B cells remained relatively quiescent in the MZ. Although generalization of our observations is limited by the nature of transgenic B cells, their different sensitivities to apoptotic cell associated antigens is consistent with the dichotomy of the TLR9 and TLR7 pathways observed in murine lupus. Two possibilities can explain why H564 B cells are more sensitive to apoptotic cells than 56R B cells. First, RNA associated antigens may distribute differently than DNA associated antigens in MFG-E8-/- mice. It is conceivable that cytosolic RNA components are directly released into the micro-environment whereas most DNA components remain insulated within intact apoptotic bodies or apoptotic blebs. H564 B cells may have easier access to their antigens than 56R B cells. Secondly, BCR/TLR9 signaling on 56R/Vκ38c B cells may inhibit their differentiation into AFCs. In fact, MFG-E8 deficiency inhibited anti-dsDNA IgG2b production by activated 56R B cells (**Fig 12B**). In a recent study by Nundel et al., using AM14 cells as a model system, the RNA-containing immune complex BW4R but not DNA-containing PA4 complex was capable of inducing AFC formation *in vitro*. More intriguingly, using an immune complex containing both TLR7 and TLR9 ligands, they demonstrated TLR9 signaling prevented TLR7 driven AFC formation [44]. Another study by Soni et al., also demonstrated the inhibitory role of TLR9 in spontaneous GC formation in autoimmune B6.SLE1b mice [45]. As 56R B cells and H564 B cells are tolerized at different stages of B cell differentiation, the interpretation of our results is confounded by the intrinsic differences between 56R and H564 B cells. However, even after LPS stimulation to release them from potential developmental arrest, 56R/Vκ38c B cells did not down-regulate their surface BCR expression after adoptive transfer (**Fig 12A**). BCR/TLR9 signaling may also actively prevent the down-regulation of BCR on their surface. Indeed, Nundel et al. found that deficiency in TLR9 allowed sustained down-regulation of BCR components *in vitro* [44]. Despite the differences between model systems, our observations provided some explanation of the protective role of TLR9 in murine lupus. The MZ B cell arrest of 56R B cells in MFG-E8-/- mice suggests that a significant portion of anti-dsDNA B cells do not progress to IgG producing AFCs in response to apoptotic cells. Instead, they are prone to produce protective IgM antibodies to facilitate the removal of apoptotic cell debris [46].
